# Household Hints and Recipes

**Published:** 1874-01

**Authors:** 


### Household Hints & Recipes.

Ether Glue.—An excellent liquid glue is made by dissolving glue in nitric ether. The ether will only dissolve a certain amount of glue, consequently the solution cannot be made too thick. The glue thus made is about the consistency of molasses, and is doubly as tenacious as that made with hot water. If a few bits of india-rubber, cut into scraps the size of buck-shot, be added, and the solution be allowed to stand a few days, being stirred frequently, it will be all the better, and will resist the dampness twice as well as glue made with water.

---

Water for Houses.—Speaking of water supply and drainage, Mr. Roberts, in a paper read before the Royal Institute of British Architects, stated that well-drained clay is a better site for a house than gravel. The explanation is, that wells dug in clay soil contain purer water than wells in gravel, for the surface water will not soak through and carry down its impurities in clay as it does in gravel. It is well known that wells in gravelly soil are liable to contamination by soakage from the surface. But the healthiness of the house, no less than the purity of the water, depends on thorough drainage of the clay.

---

Our Weights.—Upon the average, boys at birth weigh a little more, and girls a little less than six pounds and a half. For the first twelve years the two sexes continue nearly equal in weight, but beyond that time males acquire a decided preponderance. Thus, young men of twenty average about 143 lbs. each, while the young women of twenty average 120 lbs. Men reach their heaviest bulk at about thirty-five, when they average about 152 lbs.; but women slowly increase in weight until fifty, when their average is about 128 lbs. Taking men and women together, their weight at full growth averages about twenty times as heavy as they were on the first day of their existence. Men range from 108 to 220 lbs., and women from 88 to 207 lbs. The actual weight of human nature, taking the average of ages and conditions—nobles, clergy, tinkers, tailors, maidens, boys, girls, and babies all included—is very nearly 100 lbs. These figures are given in avoirdupois weight; but the advocates of the superiority of women might make a nice point by introducing the rule that women be weighed by Troy weight—like other jewels—and men by avoirdupois. The figures will then stand: young men of twenty, 143 lbs. each; young women of twenty, about 146 lbs. each (the *Sanitarian*, from which we take these figures, says "160 lbs.;" but that is not the correct equivalent for 120 lbs. avoirdupois); and so on.

---

Moth Preventative.—The following recipe for keeping moths out of clothing is a favorite in some families: Mix half a pint of alcohol, the same quantity of spirits of turpentine, and two ounces of camphor. Keep in a stone bottle, and shake before using. The clothes or furs are to be wrapped in linen, and crumpled-up pieces of blotting paper dipped in the liquid are to be placed in the box with them, so that it smells strong. This requires renewing about once a year.

---

To Purify Tallow.—Tallow when properly purified may be substituted for many purposes for which lard is now used. The fresh tallow should be melted in boiling water, and strained while hot through a linen filter. It is then again boiled with water, skimmed off, and allowed to solidify. It should be dried as thoroughly as possible by pressure, and then be again melted and run into earthen pots. When thus treated it will keep fresh for a long time.

---

Liquid Nourishment for Sick Stomach.—The *Dublin Medical Journal* commends the following: An egg, well beaten up, to which add one pint of good milk, one pint of cold water, and salt to make it palatable; let it then be boiled, and when cold any quantity of it may be taken. If it turns into curds and whey it is useless.

---

Fried Bread.—Put into a common biscuit pan a heaping teaspoonful of butter, and let it melt and spread over the pan; then take enough slices of bread (stale answers as well as any) to cover the bottom of the pan, and make a mixture to dip them in by beating well two eggs, and pouring in milk enough to soak the bread, seasoning it with a little pepper and salt; make the bread quite moist; then lay it on the butter and fry brown one side; and if too soft to turn, put it into the oven to brown on the top.

To Preserve Bread a Long Time.—Cut the bread into thick slices, and bake it in an oven, so as to render it perfectly dry. In this condition it will keep good for any length of time required. It must, however, be carefully kept from pressure; otherwise, owing to brittleness, it will soon fall to pieces. When required for use, dip the bread for an instant into warm water, and then hold it before the fire till dry; then butter it, and it will taste like toast. This is a useful way of preserving bread for voyages, and also any bread that may be too stale to be eaten in the usual way.

---

A Good Pudding.—The following recipe is specially commended by that excellent paper the *Germantown Telegraph*, of Philadelphia: Pare and core half a dozen easily cooked apples, chop them into small bits; dry some bread in the oven—stale is the best—until it is crisp, then roll it into crumbs; butter a deep dish and place in it a layer of crumbs; then put in the apples, with a little sugar, and such spices as you like; cover the apples with another layer of crumbs, and so on, adding a little beef suet, chopped as finely as possible; pour in half a pint of milk; bake till nicely browned, and serve with hard sauce.

---

Ingrowing Nails.—Dr. J. Todd, of New Orleans, sends us the following note:

In your last number you give a mode of curing ingrowing toe-nails. Allow me to say that prevention is better than cure. The cause of nails, either on the fingers or toes, growing into the flesh is that the nails are cut toward the flesh, instead of from it. To pare the nail, cut from the corner to the centre of it; and if any ingrowth has already begun, cut the centre of the nail a little hollow. By this means nature hastens to fill the vacant space, and the growth is immediately directed from the flesh, and in a short time the nail will assume a much better form, growing from, instead of into, the flesh. The nurseryman in pruning trees always cuts outward, never inward; otherwise he would make a shapeless, unprofitable tree.

---

English Salad Sauce.—Pound in a mortar the hard-boiled yolk of an egg; mix with it a saltspoonful of salt, a teaspoonful of mustard flour, a mashed meally potato, two dessert spoonfuls of good vinegar.

Ice and Cold Water as Local Applications in Scarletina, Croup, etc.—Dr. Carson, in a series of able papers in the *Philadelphia Medical and Surgical Reporter*, calls attention to the happy results following the use of ice and cold water in scarletina, diphtheria, croup, quinsy, etc. The good effects of ice-water applications to the throat externally, and ice in substance as a gargle to the throat, were almost magical. Fatality was vastly lessened, recovery was rapid, and sequelæ uncommon. The cases of scarletina were not selected, but all were given,—malignant, anginose, and simple. Dr. C's observations as to the effects of these agents run through some twenty or more years. As his experience with them has increased, so has his faith. He reasons that the diseases in question are originally local in their action, the systemic trouble being secondary. Combat and arrest the local trouble, and the systemic disorder will be prevented or lessened. He condemns the practice which consists in caustics, astringents, irritants, and poultices to the throat and neck, as but adding fuel to the fire. The indications are to cool the burning throat, to constringe and tone up the blood-vessels, and lessen the fever. All this is accomplished by allowing the patient to suck ice, to gargle the throat with ice-water, and by enveloping the neck in bags of ice, and by cold affusions to the general surface. This practice is to be kept up steadily throughout the whole course of scarletina. This, together with support by nourishment, is all that need be done in the mildest as well as the severest cases. Albuminuria and enlarged glands do not contra-indicate the use of ice and ice-water. Dr. C. contends that there is no danger of retrocession of the disease under the use of these agents. He gives over one hundred cases of scarletina in one year, all treated by these means, with not a single fatal result.

The same treatment is equally successful in diphtheria, membranous croup, and quinsy. It is remarkable that our text-books on practice and the diseases of children have given hardly a passing notice to these simple therapeutics in the management of diseases so dangerous to life.

Glycerine Hair Wash.—Glycerine, 2 fluid ounces; water, 6 fluid ounces; oil of rosemary, 6 drops. Mix together. This is an excellent remedy against dandruff.

Welsh Rarebit.—Put into a frying-pan a quarter of a pound of cheese cut up into thin slices. Pour on it half a pint of sweet milk. Stir in an egg that was already beaten up, add a fourth of a teaspoonful of mustard, a little red pepper already ground, and a teaspoonful of nice butter. Stir this mixture all the time. Then add, lastly, a few crackers well broken up, and after thoroughly incorporating them into the mixture, turn it all into a heated dish and cover it.

---

Uses of Rawhide.—The skin of an animal, whether cow, calf, colt, or horse, that dies on the farm, is worth more at home than at the tanner's. Cut it into narrow strips, and shave off the hair with a sharp knife before the kitchen fire, or in your workshop, on stormy days and evenings. You may make them soft by rubbing. A rawhide halterstrap an inch wide, will hold a horse better, and last longer, than an inch rope. It is stronger than hoop-iron and more durable, and may be used to hoop dry casks and boxes, and for hinges.

Try it on a broken thill, or any wood-work that has been split. Put it on wet, and nail fast. Thin skins make the best bag-strings in the world. A rawhide rope is a good substitute for a chain. It is valuable to mend a broken link in a trace-chain. For some purpose it is best to use it in its natural state. For other purposes it may be dressed soft.

---

Turpentine in Headache.—Dr. Warburton Begbie (*Edinburgh Medical Journal*) advocates the use of turpentine in the severe headache to which nervous and hysterical women are subject. "There is, moreover," he says, "another class of sufferers from headache, and this is composed of both sexes, who may be relieved by turpentine. I refer to the frontal headache, which is most apt to occur after prolonged mental effort, but may likewise be induced by unduly-sustained physical exertion,—what may be styled the headache of a fatigued brain. A cup of very strong tea often relieves this form of headache; but this remedy, with not a few, is perilous, for, bringing relief to pain, it may produce general restlessness and—worst of all—banish sleep. Turpentine, in doses of twenty or thirty minims, given at intervals of an hour or two, will not only remove the headache, but produce in a wonderful manner, that soothing influence to which reference has already been made."

Carpeted Floors.—When a carpet is taken up to be cleaned, the floor beneath it is generally very much covered with dust. This dust is very fine and dry, and poisonous to the lungs. Before removing it, sprinkle the floor with very dilute carbolic acid, to kill any poisonous germs that may be present, and to thoroughly disinfect the floor and render it sweet.

---

Chloral in Toothache.—Dr. Page, in the *British Medical Journal*, states that for some time past he has employed chloral hydrate, not as an internal sedative in dental neuralgia and caries, but also as a local application to a carious tooth. A few grains of the solid hydrate placed on a quill-point, introduced into the dental cavity, speedily dissolve, and the pain is either deadened or effectively allayed. A second or third application of the remedy may be necessary.

---

"Breaking in" Boots and Shoes.—A writer in the *Oneida Circular* concludes a discourse on this text with the following "practical remarks," which we heartily endorse:

1. Never "break in" boots or shoes. If they are not easy when new, don't take them; for the boots will break your feet oftener than your feet will break the boots.

2. If you go on "breaking in" boot leather, you will need a epecial last, made with all sorts of knobs and protuberances to correspond with your distorted joints. Then you will be sorry.

3. If you have large feet, admit it all in honesty, and have your boots made accordingly. Then you will be happy.

---

Moths.—In India, both upholsterers and saddlers were badly troubled with moths in their work, especially in the rainy season; and the upholsterers in that country follow a series of simple rules by which they entirely avoid the ravages of these pests. They never put on a burlap or cotton covering without first steeping it in a solution of sulphate of copper, made by dissolving about one ounce in one gallon of boiling water, and then quickly drying the material in the sun or by a hot stove. For over coverings, especially if of wool, a solution of corrosive sublimate dissolved in patent colorless alcohol, is frequently used with good effect. The boiling solution of sulphate of copper is often applied to a floor previous to laying a mat or carpet, and invariably under heavy articles of furniture.

Fish as a Diet.—Dr. Merryweather says: "A fish diet is a great humanizer of the tempers of mankind. Its consumption tends wonderfully to render them more kindly to one another, and consequently tames the passionate disposition to crime. As carnivorous animals are always the most fierce and violent, so become human beings who have carnivorous stomachs. Could such stomachs have an occasional respite by the consumption of fish, the world would be all the better for it. I speak as a medical man, and firmly assert that many maladies would be mitigated, and perhaps annihilated by such a process."

---

To Clean Looking-Glasses.—Take a newspaper, fold it small, dip it in a basin of clean cold water. When thoroughly wet, squeeze it out as you do a sponge; then rub it pretty hard all over the surface of the glass, taking care that it is not so wet as to run down in streams; in fact, the paper must only be completely moistened, or dampened, all through. Let it rest a few minutes, then go over the glass with a piece of fresh newspaper, till it looks clear and bright. The insides of windows may be cleaned in the same way; also spectacle glasses, lamp-glasses, etc. White paper that has not been printed on is better; but in the absence of that a very old newspaper, on which the ink has become thoroughly dried, should be used. Writing paper will not answer.

---

Orange-Peel Poisonous.—The *Pacific Medical and Surgical Journal* says:—Now that oranges are in every child's mouth,it is well for parents to know that fatal consequences may follow the swallowing of the rind. Many years ago we had in charge two little girls, sisters, four and six years of age, who were seized with violent inflammation of the bowels from this cause. One of them died in convulsions, and the other had a narrow escape. Since that time quite a number of instances, similiar in character, have come under our observation.

Quite recently we saw a child something over a year old, that was attacked with violent dysenteric symptons, for which no cause could be assigned. The attack came on during the passage of the steamer from San Diego. The symptoms were so identical with those with which we had previously noticed to arise from poisoning by orange-peel, that we were induced to inquire particularly if the child had had an oppurtunity of getting this substance into its mouth. We were informed that it had been playing with an orange, and nibbling at it, just before the attack of the disease. The discharge from the bowels were frequent, and consisted of blood and mucus. After a week of severe enteric inflammation, the child died. We have no doubt that the disease was brought on by the rind of orange. A small quantity of such an indigestible and irritating substance will often produce most serious consequences.

---

Hygienic Use of Tea.—Dr. Adam Smith, in a paper read before the London Society of Arts, recommends the use of tea in the following cases: After a full meal, when the system is oppressed; for the corpulent and the old; for hot climates, and especially for those who, living there, eat freely, or drink milk or alcohol; in cases of suspended animation; for soldiers who, in time of peace, take too much food in relation to the waste proceeding in the body; for soldiers and others marching in hot climates, for then, by promoting evaporation and cooling the body, it prevents in a degree the effects of too much food, as of too great heat.

---

Toilet Vinegar.—The London *Chemist and Druggist* recommends the following formula for this popular toilet article:

| | |
|---|---|
| Ess. bergamot - - - | 20 min: |
| " ambergris - - - - | 4 dr. |
| " vanilla - - - - | 30 min. |
| Ol. neroli - - - - - | 30 min. |
| Acid acetic - - - - | 140 min. |
| Spirit vini rect, - - - - | 5 oz. |
| | Mix. |

---

Glycerine Hair Tonic.—Glycerine, 2 fluid ounces; alcohol deoderized, 12 fluid ounces; castor oil, 3 fluid ounces; oil of rosemary, 20 drops, or any other purfume. Dissolve the castor oil and oil of rosemary in the alcohol and add gradually the glycerine.

---

A New Substitute for Rubber. —A Canadian has devised a method of producing gum from the milkweed plant, or other plants of the asclepia family, and flax and other seeds, which consists in macerating and fermenting the substances, and then, by evaporation, reducing the resulting liquid to a thick, gummy mass. The gum thus obtained may be cheaply produced, and is alleged to have many of the valuable qualities of rubber. It is insoluble in water, and may be vulcanized with sulphur, etc.

